# Women Benefit More Than Men in Response to College-based Meditation Training

**DOI:** 10.3389/fpsyg.2017.00551

**Published:** 2017-04-20

**Authors:** Rahil Rojiani, Juan F. Santoyo, Hadley Rahrig, Harold D. Roth, Willoughby B. Britton

**Affiliations:** ^1^Yale School of Medicine, New HavenCT, USA; ^2^Contemplative Studies Initiative, Brown University, ProvidenceRI, USA; ^3^Department of Neuroscience, Brown UniversityProvidence RI, USA; ^4^Department of Psychiatry and Human Behavior, Warren Alpert Medical School of Brown University, ProvidenceRI, USA; ^5^Department of Religious Studies, Brown University, ProvidenceRI, USA

**Keywords:** mindfulness, meditation, gender, emotion, affect

## Abstract

**Objectives:** While recent literature has shown that mindfulness training has positive effects on treating anxiety and depression, there has been virtually no research investigating whether effects differ across genders—despite the fact that men and women differ in clinically significant ways. The current study investigated whether college-based meditation training had different effects on negative affect for men and women.

**Methods:** Seventy-seven university students (36 women, age = 20.7 ± 3.0 years) participated in 12-week courses with meditation training components. They completed self-report questionnaires of affect, mindfulness, and self-compassion before and after the course.

**Results:** Compared to men, women showed greater decreases in negative affect and greater increases on scales measuring mindfulness and self-compassion. Women’s improvements in negative affect were correlated to improvements in measures of both mindfulness skills and self-compassion. In contrast, men showed non-significant increases in negative affect, and changes in affect were only correlated with ability to describe emotions, not any measures of experiential or self-acceptance.

**Conclusion:** These findings suggest that women may have more favorable responses than men to school-based mindfulness training, and that the effectiveness of mindfulness-based interventions may be maximized by gender-specific modifications.

## Introduction

Meditation is an increasingly popular form of mental training encompassing a variety of practices that aim to improve specific psychological capacities like attentional and emotional self-regulation (Tang et al., 2015). One of the most common meditation practices, mindfulness meditation, can be described as a practice of intentionally and non-judgmentally directing one’s attention to the present moment, often by focusing on a particular salient sensation, such as one’s breath (Kabat-Zinn, 1990). Mindfulness training, incorporated in clinical interventions through mindfulness-based interventions (MBIs) have been recognized as a viable treatment to prevent recurrent depression and depressive relapse (Teasdale et al., 2000; Ma and Teasdale, 2004; Britton et al., 2005; Segal and Williams, 2012) and as a potential preventative for such disorders, helping to counter behaviors such as rumination that precede and co-occur with depression (Broderick, 2005; Brown et al., 2010). More research is investigating the potential for applying MBIs in treating anxiety and stress (Chang et al., 2004; Semple et al., 2005), addiction/substance use disorder (Speca et al., 2000; Brewer et al., 2009), ADHD (Zylowska et al., 2008), and even schizophrenia (Zylowska et al., 2008; Chien and Lee, 2013).

School-based mindfulness programs have been hypothesized to be a potentially effective avenue to disseminate preventative treatment to pre-clinical populations (e.g., the MYRIAD program out of Oxford Mindfulness Centre). They are also growing in popularity as initial studies on mindfulness in school settings suggest increased resilience to emotional stress (Deyo et al., 2009), reduced emotional reactivity (Mendelson et al., 2010), increased attention, reduced behavioral problems, and improved academic performance in students (Benson et al., 2000; Napoli et al., 2005; Franco et al., 2010) although this research is limited by poor methodological rigor (Davidson et al., 2012; Greenberg and Harris, 2012).

### Gender in Mental Health: Internalizing versus Externalizing

The past several decades have produced a large body of research suggesting that men and women exhibit different trajectories of psychological symptomology. This divergence between men and women begins to appear in early adolescence during which the incidence of psychological disorders increases for both groups (Beauchemin et al., 2008), but increases more for women such that by age 13–14, girls are about twice as likely as boys to suffer from depression and anxiety (Kovacs, 1996; Angold et al., 1998; Saluja et al., 2004). Conversely, males are more likely to have conduct disorder and substance use disorder (Helzer et al., 1991; Lahey et al., 1999; Cotto et al., 2010). This pattern persists throughout adulthood (Nolen-Hoeksema, 1994) and has shown to be a relatively cross-cultural phenomenon (Weissman et al., 1996; Kuehner, 2003).

Importantly, research also suggests differences in response mechanisms to psychological distress. When coping with psychological distress, men tend to “externalize” their distress by directing action outward (e.g., playing sports or video games, watching TV, etc), whereas women tend to internalize their distress by directing action inward (e.g., ruminating or writing about a negative event) (Broderick, 1998; Li et al., 2006). This difference in “internalizing” versus “externalizing” emotional regulation strategies continues throughout adulthood (Kleinke et al., 1982; Matud, 2004). Further, this difference may mediate the divergence in psychological symptoms that emerges during adolescence (Nolen-Hoeksema, 1994; Kraaij et al., 2003; Kramer et al., 2008), thus resulting in men with more “externalizing disorders” like conduct disorder and substance use disorder, and women with more “internalizing disorders” like depression and anxiety.

### The Potential Role for Mindfulness Training

Since mindfulness training may improve psychological health by influencing how individuals cope with their emotions (Weinstein et al., 2009), it seems possible that this type of mental health treatment may yield contrasting effects in groups with different coping strategies at baseline. Divergent gender effects of movement-based meditation were observed in a study on the efficacy of qigong meditation in residential addiction treatment which found that, following treatment, women in their sample reported more reduction in anxiety and withdrawal symptoms than men (Chen et al., 2010). Additionally, a review of MBIs in the treatment of substance use disorder reported divergent gender responses to interventions, with women preferring and benefiting more from mindfulness treatments (Katz and Toner, 2013). Despite this initial work on divergent gender effects in addiction and substance use contexts specifically, broader divergent gender effects in MBIs is currently under-investigated.

The current study investigated divergent gender effects in school-based meditation program outcomes in college students, with a particular focus on self-reported changes in affect, self-compassion and mindfulness and the mechanisms underlying these changes. We hypothesized that women would have greater affect disturbance at baseline and therefore greater improvement in affect (both positive and negative) than their male counterparts. We predicted that these improvements in affect would counteract gender-specific coping methods. Specifically, we expected that women’s improvements in affect would be associated with increased self-acceptance while men’s improvements in affect would be associated with decreased distraction.

## Materials and Methods

### Participants

Participants (*N* = 77, 36 women, age = 20.7 ± 3.0 years) included Brown University undergraduates enrolled in 12 week courses with meditation training components. Courses included both weekly didactic seminars as well as experiential practice-based learning through “meditation labs” described below.

Participants were recruited in the first week of classes. Study staff came to class to describe the study and circulated sign-up sheets. Students who wanted to participate and were at least 18 years old completed written informed consent procedures. The study protocol was approved by the Brown University Institutional Review Board.

Of the 114 students (54 women) who started the study, 37 participants (18 women) were excluded from analysis due to lack of adequate participation, incomplete questionnaires, or loss to follow up. This resulted in a 32% rate of attrition.

### Procedures

Participants completed questionnaires at the beginning and end of the 12-week course. These courses took place during each college semester between January 2008 and May 2011 on the Brown University campus in Providence, Rhode Island. Adverse events were monitored with seven critical items of the Brief Symptom Inventory (BSI) (Derogatis and Melisaratos, 1983) related to suicidality, panic and violence. Study staff reviewed the BSI, and endorsements of any of the critical items resulted in an in-person query by a clinical psychologist and referrals to Brown University psychological services, if necessary.

### Measures

#### Affect

The Positive and Negative Affect Scale (PANAS; Watson et al., 1988) was used to assess current affective states. This 20-item scale asks participants to describe the degree to which they are experiencing particular affective states in the present moment, with 10 items measuring positive affect (e.g., interested, excited, enthusiastic) and 10 measuring negative affect (e.g., ashamed, distressed, irritable). Responses range from 1 (not at all) to 5 (extremely). The PANAS was previously shown to have high reliability and validity (Crawford and Henry, 2004) and showed good internal consistency at both pre (α = 0.78) and post (α = 0.80) timepoints.

#### Mindfulness

The Five Facet Mindfulness Questionnaire (FFMQ; Baer et al., 2006) was used to assess mindfulness skills. This 39-item inventory has five subscales designed to assess different aspects of mindfulness: observing (α = 0.76; α = 0.80), describing (α = 0.88; α = 0.89), acting with awareness (α = 0.87; α = 0.89), accepting without judgment (α = 0.89; α = 0.92), and non-reactivity to inner experience (α = 0.86; α = 0.86). Total score internal consistency was high at both timepoints, α = 0.88, α = 0.89. Participants were asked to rate how often certain statements described themselves from each of the five dimensions of observing (e.g., “When I’m walking, I deliberately notice the sensations of my body moving”), describing (e.g., “I’m good at finding words to describe my feelings”), acting with awareness (e.g., “I do jobs or tasks automatically without being aware of what I’m doing,” reverse-scored), accepting without judgment (e.g., “I tell myself I shouldn’t be feeling the way I’m feeling,” reverse-scored), and non-reactivity to inner experience (e.g., “I watch my feelings without getting lost in them”). Responses ranged from 1 (“never or very rarely true”) to 5 (“very often or always true”). The FFMQ was previously shown to have high reliability and validity (Baer et al., 2008; Christopher et al., 2012).

#### Self-compassion

The Self-Compassion Scale (SCS; Neff, 2003) was used to assess levels of kindness and acceptance toward oneself. This 26-item assessment measures six subscales that are paired and scored as if expressed in diametric opposition to each other: self-kindness versus self-judgment, common humanity versus isolation, and mindfulness versus over-identification. Participants were asked to rate the frequency of certain behaviors corresponding to the six different subscales of self-kindness (e.g., “I try to be understanding and patient toward those aspects of my personality I don’t like”), self-judgment (e.g., “I’m disapproving and judgmental about my own flaws and inadequacies”), common humanity (e.g., “I try to see my failings as part of the human condition”), isolation (e.g., “When I fail at something that’s important to me, I tend to feel alone in my failure”), mindfulness (e.g., “When something upsets me I try to keep my emotions in balance”), over-identification (e.g., “When I’m feeling down, I tend to obsess and fixate on everything that’s wrong”). Responses range from 1 (“almost never”) to 5 (“almost always”). The SCS inventory was found to have high internal consistency (α = 0.91; α = 0.93). The SCS was previously reported to have high reliability and validity (Neff, 2003; Costa et al., 2015).

#### Meditation Practice Amount

Mediation practice amount was calculated based on attendance at thrice weekly 1-h lab sessions (36 total), attendance at optional weekend retreats, and a questionnaire that asked about the number of practices sessions attended per week and duration of each session outside of class.

### Meditation Labs

Meditation labs were scheduled for 1 h three times per week and included approximately 30 min of a specific contemplative practice from Buddhist or Daoist traditions. The meditation laboratory was taught by Professor Harold Roth, a published scholar of Buddhist and Daoist contemplative practices with more than 35 years of personal practice experience in the Rinzai Zen tradition. The meditation period was followed by a 5–10 min written reflection period and question-and-answer period.

Meditation training involved primarily focused attention and open-monitoring forms of practice, including focused attention training on a single object (like the breath) or a class of objects (like body sensations). Meditation instruction emphasized attention allocation rather than the non-judgmental acceptance that is common in more modern Western styles of mindfulness, although it did incorporate instructions to refrain from mental evaluation of experience.

### Analyses

#### Preliminary Analyses

Before analysis, all variables were examined for normality. Winsorized cases included two participant’s (both men) values for post-intervention PANAS negative affect which were high outliers and were replaced with the next highest non-outlying value (Cohen, 2001). Cases with missing data were excluded from analysis. Preliminary analyses were used to describe baseline characteristics, differences in time spent meditation by group and participant flow/adherence, as well as to investigate any baseline group differences that might affect the main analyses.

#### Main Analysis

The main analysis investigated the degree of change on self-report measures in women compared to men. Separate two-way repeated measures analyses of variance (ANOVA) were conducted on the PANAS positive and negative affect scales and the individual subscales of the FFMQ and SCS. Variables were two-level within-subject measures (pre and post). The between-subjects variable was gender (men, women). For all scales and subscales, the time effects and time × gender effects are reported in the text and in **Table 1**. Paired *t*-tests were also conducted on all scales and subscales to assess within-group effects of the training, i.e., effect size of pre–post changes in men and effect size of pre–post changes in women. These are reported in the text for PANAS and FFMQ subscales for which there were significant time × gender interactions, as well as for all SCS subscales. Non-significant effects and other results are also reported in **Table 1**.

**Table 1:** Mean (SD) measures before and after meditation training in women and men, with analysis of variance results.

				*F*(1,76)		
Measures	Men	(*n* = 41)	Women (*n* = 36)	Time	Gender	Time × Gender
**Demographics**					
Age (Years)	20.53 (3.26)	20.83 (2.62)	0.41		
**Affect (PANAS)**					
Baseline positive affect	33.63 (5.45)	34.50 (6.60)	1.91	0.77	0.115
Exit positive affect	33.71 (6.36)	35.08 (7.52)			
Baseline negative affect	19.10 (5.95)	21.11 (6.24)	2.73	0.12	9.00^∗∗^
Exit negative affect	19.80 (5.71)	18.67 (6.05)^∗∗^			
**Mindfulness (FFMQ)**					
Baseline observe	28.46 (4.86)	27.0 (4.57)	18.13^∗∗∗^	0.11	6.76^∗^
Exit observe	29.20 (4.20)	30.03 (4.66)^∗∗∗^			
Baseline describe	26.78 (5.69)	27.44 (5.47)	22.86^∗∗∗^	2.43	8.84^∗∗^
Exit describe	27.51 (5.91)	30.58 (4.91)^∗∗∗^			
Baseline act-aware	24.95 (4.48)	23.36 (4.70)	11.41^∗∗∗^	1.12	1.47
Exit act-aware	26.00 (4.20)	25.58 (5.27)^∗∗^			
Baseline non-judge	28.17 (5.80)	26.22 (6.19)	33.55^∗∗∗^	0.27	5.23^∗^
Exit non-judge	30.29 (5.02)^∗∗^	31.11 (4.57)^∗∗∗^			
Baseline non-react^∗∗^	22.00 (4.31)	19.06 (4.32)	70.63^∗∗∗^	5.02^∗^	5.71^∗^
Exit non-react	24.59 (4.27)^∗∗∗^	23.69 (3.82)^∗∗∗^			
Baseline total^∗^	130.3 (16.4)	123.08 (14.93)	65.63^∗∗∗^	0.37	11.89^∗∗^
Exit total	137.5 (15.5)^∗∗∗^	141.0 (14.28)^∗∗∗^			
**Self-Compassion (SCS)**					
Baseline self-kindness	15.12 (3.78)	14.39 (3.54)	40.35^∗∗∗^	0.48	0.302
Exit self-kindness	17.32 (3.59)^∗∗∗^	17.00 (3.91)^∗∗∗^			
Baseline common humanity	11.83 (3.32)	11.36 (3.04)	13.73^∗∗∗^	0.02	2.15
Exit common humanity	12.68 (3.64)	13.33 (3.88)^∗∗∗^			
Baseline mindfulness	13.58 (2.81)	13.06 (2.34)	21.59^∗∗∗^	0.84	0.03
Exit mindfulness	14.9 (2.51)^∗∗^	14.47 (2.81)^∗∗∗^			
Baseline self-judgment	15.51 (3.59)	14.19 (4.21)	21.51^∗∗∗^	0.31	3.69
Exit self-judgment	16.73 (4.05)^∗^	17.14 (4.52)^∗∗∗^			
Baseline isolation	12.34 (3.77)	12.14 (3.31)	24.38^∗∗∗^	0.11	1.92
Exit isolation	13.51 (3.34)^∗∗^	14.22 (3.93)^∗∗∗^			
Baseline over-Identification	12.76 (3.44)	12.17 (3.39)	31.07^∗∗∗^	0.06	1.98
Exit over-identification	14.00 (3.20)^∗∗^	14.25 (3.65)^∗∗∗^			
Baseline total	81.15 (2.36)	77.31 (2.39)	48.82^∗∗∗^	0.16	2.86
Exit total	89.16 (2.33)^∗∗∗^	90.42 (2.89)^∗∗∗^			

*Mean values are displayed with standard deviations in parentheses where applicable. Asterisks in column 1 (Measures) indicate measures with significant baseline differences between men and women. Asterisks in columns 2 and 3 (Men and Women) indicate significant within-gender pre-post changes, i.e., for men [*t*(40)] and women [*t*(35)] separately. Asterisks in columns 4, 5, and 6 (Time, Gender, Time × Gender) indicate significant results in the analysis of variance (ANOVA). ^∗^*p* < 0.05, ^∗∗^*p* < 0.01, ^∗∗∗^p < 0.001*.

#### Secondary Analysis

Pearson product-moment correlation coefficients were used to assess the relationship between changes in affect (as measured by the PANAS) and changes on mindfulness and self-compassion scores (as measured by the FFMQ and SCS) for women and men respectively. Data were analyzed using SPSS 19.0 software (SPSS Inc, Chicago, IL, USA). Statistical significance was set at alpha levels <0.05, two-tailed. Results are reported as mean ± SD unless otherwise indicated. Effect sizes are reported as partial η^2^ (small = 0.01, medium = 0.06, large = 0.14; Green and Salkind, 2005). Results from this analysis are reported in **Table 2**.

**Table 2:** Pearson product-moment correlations between pre–post changes in negative affect and other measures split by gender.

	Negative affect (PANAS Negative)	
Measures	Men (*n* = 41)	Women (*n* = 36)
FFMQ- Observe	-0.162	-0.132
FFMQ- Describe	-0.384^∗^	-0.139
FFMQ- Act-aware	-0.021	-0.333^∗^
FFMQ- Non-judge	-0.065	-0.326^∧^
FFMQ- Non-react	-0.164	-0.363^∗^
FFMQ Total	-0.271	-0.381^∗^
SCS- Self-kindness	-0.055	-0.244
SCS- Common humanity	-0.199	0.201
SCS- Mindfulness	-0.301	-0.083
SCS- Self-judgment	-0.231	-0.357^∗^
SCS- Isolation	-0.208	-0.003
SCS- Over-identification	-0.173	-0.508^∗∗^

*FFMQ, Five Facets of Mindfulness Questionnaire; SCS, Self-Compassion Scale. Values indicate the corresponding Pearson product-moment correlation coefficient. ^∧^*p* < 0.06, ^∗^*p* < 0.05, ^∗∗^*p* < 0.01*.

#### Adverse Event Monitoring

A total of 11 (14%) students reported some level of suicidal ideation (SI) during the study (two women, nine men). Eight students endorsed SI at baseline, two women and six men, while six students endorsed SI at the end of meditation course, all men. In terms of change over time, two women and three men reported SI at baseline but not exit, and three men developed SI during the course of the semester that was not present at baseline. These participants were not removed from analysis.

## Results

### Preliminary Analyses

#### Participant Flow

Seventy-seven individuals (36 women) who completed all baseline and post-treatment testing and attended at least 50% of the meditation labs were included in analysis.

#### Intervention Adherence

The participants reported meditating an average of 2,495 min over the course of the semester (including personal practice outside of the meditation labs; range 810–6,591 min). Men reported an average of 2,700 min and women an average of 2,266. This difference was not significant [*t*(75) = -1.66, *p* = 0.10].

#### Baseline Characteristics

Contrary to predictions that women would show more signs of affective disturbance, there were no significant differences between genders at baseline on PANAS positive affect [*t*(75) = 0.63, *p* = 0.53] or negative affect [*t*(75) = 1.45, *p* = 0.15]. Approximately 60% of the sample scored below clinical cut-offs (Watson and Clark, 1994) on the negative PANAS subscale (scores of 21.1 and 23.7 for men and women, respectively), which suggests that a minority of the sample had clinical levels of negative affect. Women scored significantly lower than men on the FFMQ non-reactivity subscale [*t*(75) = -2.99, *p* = 0.004], which also impacted the FFMQ total score [*t*(75) = -2.02, *p* = 0.047]. There were no other significant differences between genders on mindfulness skills or self-compassion at baseline.

### Main Analyses

#### Affect, Time Effects

Over time, the group as a whole demonstrated no significant changes in positive affect [*F*(1,75) = 0.19, *p* = 0.66] or negative affect [*F*(1,75) = 2.73, *p* = 0.10].

#### Affect, Time × Gender Interaction Effects

Our ANOVA analysis demonstrated significant time × gender interaction effect for negative affect [*F*(1,75) = 9.00, *p* = 0.004] which indicated that women showed a greater reduction in negative affect than their male counterparts.

#### Affect, Within Gender Effects

**FIGURE 1:**
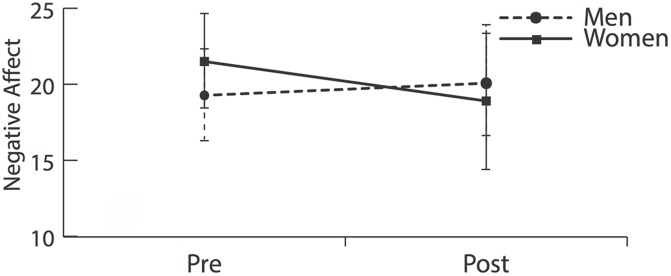
**Negative affect before and after meditation training**. Women demonstrated significantly greater reductions in negative affect than men [*F*(1,75) = 9.00, *p* = 0.004].

Paired *t*-tests indicated that women showed significant decreases [*t*(35) = 2.94, *p* = 0.006] while men showed non-significant increases [*t*(40) = -1.07, *p* = 0.29] (**Figure 1** and **Table 1**).

#### Mindfulness, Time Effects

Whole group paired *t*-tests demonstrated significant time effects indicating whole-group improvements on all 5 subscales of the FFMQ including FFMQ total [*F*(1,75) = 65.63, *p* < 0.001], FFMQ observe [*F*(1,75) = 18.13, *p* < 0.001], FFMQ describe [*F*(1,75) = 22.87, *p* < 0.001], FFMQ non-react [*F*(1,75) = 70.63, *p* < 0.001], FFMQ non-judge [*F*(1,75) = 33.55, *p* < 0.001] and FFMQ act-aware [*F*(1,75) = 11.41, *p* < 0.001].

#### Mindfulness, Time × Gender Interaction Effects

Our ANOVA analysis demonstrated significant time × gender interactions for FFMQ total [*F*(1,75) = 11.89, *p* = 0.001], FFMQ observe [*F*(1,75) = 6.76, *p* = 0.011], FFMQ describe [*F*(1,75) = 8.84, *p* = 0.04], FFMQ non-react [*F*(1,75) = 5.71, *p* = 0.019], and FFMQ non-judge [*F*(1,75) = 5.23, *p* = 0.025] indicating that women increased more than men on 4 out of 5 mindfulness subscales on the FFMQ.

#### Mindfulness, Within Gender Effects

Paired *t*-tests demonstrated that women had significant increases for FFMQ total [*t*(35) = -6.59, *p* < 0.001], FFMQ observe [*t*(35) = -4.53, *p* < 0.001], FFMQ describe [*t*(35) = -5.68, *p* < 0.001], FFMQ non-react [*t*(35) = -6.36, *p* < 0.001], and FFMQ non-judge [*t*(35) = -4.44, *p* < 0.001]. On the other hand, men demonstrated smaller but also significant increases on FFMQ total [*t*(40) = -4.35, *p* < 0.001], FFMQ non-react [*t*(40) = -5.29, *p* < 0.001], and FFMQ non-judge [*t*(40) = -3.57, *p* = 0.001]. Men showed no significant change in FFMQ observe or FFMQ describe.

#### Self-compassion, Time Effects

Whole group paired *t*-tests demonstrated significant time effects for SCS total [*F*(1,75) = 48.82, *p* < 0.001] indicating increases in self-compassion for both genders. Further, a significant time effect for SCS self-kindness [*F*(1,75) = 40.35, *p* < 0.001], SCS self-judgment [*F*(1,75) = 21.50, *p* < 0.001], SCS common humanity [*F*(1,75) = 13.73, *p* < 0.001], SCS isolation [*F*(1,75) = 24.37, *p* < 0.001], SCS mindfulness [*F*(1,75) = 21.59, *p* < 0.001], and SCS over-identification [*F*(1,75) = 31.10, *p* < 0.001] indicated a general increase on all subscales across all participants.

#### Self-compassion, Time × Gender Interaction Effects

Our ANOVA analysis demonstrated no significant time × gender interaction effect for SCS total or any SCS subscales.

#### Self-compassion, Within Gender Effects

Paired *t*-tests demonstrated that women and men both showed significant pre–post improvements on SCS total [women *t*(35) = -6.59, *p* < 0.001; men *t*(40) = -5.15, *p* < 0.001], all six subscales for women, and all subscales except SCS common humanity for men (**Table 1**).

### Secondary Analyses, Correlations with Changes in Negative Affect

In women, decreases in PANAS negative affect were significantly correlated with increases in FFMQ total, FFMQ act-aware, FFMQ non-react, SCS self-judgment, SCS over-identification, and at trend-level in FFMQ non-judgment (**Table 2**).

In men, decreases in PANAS negative affect were significantly correlated with improvements on FFMQ describe, but were not significantly correlated with any other scales (**Table 2**).

## Discussion

Our results suggest that women benefited more from school-based mindfulness training than men: Women showed significant reductions in negative affect while men showed slight but non-significant increases (**Figure 1** and **Table 1**). Affect-related gender differences were specific to negative and not positive affect—neither gender showed increases in positive affect.

**FIGURE 2:**
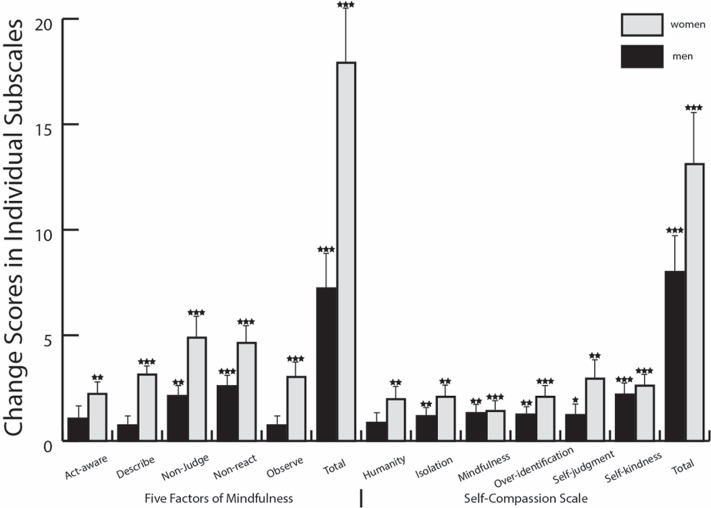
**Pre to post changes by gender in measures of mindfulness and self-compassion**. ^∗^*p* < 0.05, ^∗∗^*p* < 0.01, ^∗∗∗^*p* < 0.001. Stars indicate significant pre–post changes for each gender individually. Error bars represent SEM.

Additionally, women increased more than men on domains of mindfulness involving non-reactivity, non-judgment, observing emotions (**Table 1**). However, compared to their respective baselines, both women and men improved significantly in most measures (thirteen and nine, respectively; **Figure 2**). Correlations became the key distinguishing factor.

For women, decreases in negative affect were significantly correlated with improvements in mindfulness skills involving their tendency to notice thoughts and emotions without identifying with or judging them, and self-compassion skills involving increased self-kindness and reduced tendencies toward self-judgment and over-identification with emotions. In contrast, men’s improvements on mindfulness and self- compassion did not correlate with improvements in negative affect. This suggests that the therapeutic mechanism of mindfulness for negative affect may be gender-specific.

### Why Are Women Improving More Than Men?

Our research finding that women in this sample are benefiting more than men is consistent with previous studies on mindfulness-based treatment for substance use disorders (Chen et al., 2010; Katz and Toner, 2013). In determining why this might be true, our analysis excluded the important potential explanations of baseline differences or differences in hours meditated. In particular, if men and women started with different levels of affect at baseline, there might be more room and likelihood for improvement. However, there were no baseline differences in positive or negative affect (**Table 1**). Further, reported hours show that on average, men actually meditated over 7 h more than women over the course of the 12-week semester (though this difference was not statistically significant; see Intervention Adherence). Thus, women did not improve more than men by meditating more and getting a higher “dose” of treatment.

We suggest that the divergent effects we observed were caused primarily by gender-based mechanistic differences in emotion regulation techniques, which have also been reported in other contexts. Gendered differences in emotion regulation techniques have been indicated by neuroimaging research using fMRI which has reported that men demonstrate less activation of brain regions involved in emotional regulation (amygdala, prefrontal regions associated with emotion regulation, and reward-associated ventral striatal regions) during active emotional regulation (McRae et al., 2008). A similar fMRI study found that negative emotion induction during working memory tasks results in greater recruitment of emotion-association regions (amygdala and the orbitofrontal cortex) in women whereas in men, regions associated with cognitive control (prefrontal and superior parietal regions) remain more activated (Koch et al., 2007).

The difference in mechanisms of emotion regulation is also supported by research reporting that men and women have divergent behavioral responses to negative situations and emotions (Lighthall et al., 2009); women tend to “internalize” by ruminating or engaging in self-critical behavior, while men tend to “externalize” by distracting themselves or engaging the environment (Broderick, 1998; Li et al., 2006; Johnson and Whisman, 2013).

Drawing on other studies which suggest that mindfulness interventions decrease rumination (Jain et al., 2007; Kingston et al., 2007; Shahar et al., 2010; Heeren and Philippot, 2011), we propose the hypothesis that mindfulness interventions may produce better results for women by decreasing ruminative tendencies which targets women’s tendencies toward an internalized response to distress. Conversely, increased attention toward (rather than away from) thoughts and emotions in men may result in increased negative affect.

Our data corresponds well with this hypothesis. The correlations we observed suggest that improved affect in women was related to improved mindfulness and self-compassion skills, which involved specific subscales for approaching experience and emotions with non-reactivity, being less self-critical and more kind with themselves, and over-identifying less with emotions. These observations support elements of one theoretical explanation for how mindfulness might decrease rumination: a two-factor pathway in which attentional clarity and acceptance of experience contribute to a clarity of experience (Bishop et al., 2004) and eventually to improved negative emotion regulation strategies (Bishop et al., 2004; Coffey et al., 2010).

In contrast, while men in our sample also showed improvements in measures of non-reactivity, non-judgment, and self-compassion, they showed no overall changes in negative affect. To the extent that affect improved, changes were correlated with an improved dimension of mindfulness involving the ability to identify, describe and differentiate one’s emotions. We propose that this relationship did not yield a significant improvement in affect as an increased exposure to previously ignored emotions could have buffered improvements in mood or even have a negative effect. Further research should investigate this hypothesis.

If improvement in affect in men are associated with the ability to describe and differentiate emotions, then practices that emphasize this skill, such as open-monitoring and affect labeling may be more beneficial to men. Recent research has developed 8-week MBIs that teach advanced skills in open monitoring and labeling of ongoing experience (Britton, 2011). It is also possible that more active methods of mindfulness training, such as yoga or Taiji (Tai Chi), may work better for men rather than silent meditation training without movement, given that they may better accommodate the external coping strategy more typical of men. Previous research has already indicated that men and women with higher masculinity ratings respond better to physically active forms of stress reduction (Friedman and Berger, 1991), substantiating a utility for such targeted treatment.

### Why Improvements in Negative Affect But Not Positive Affect?

Our data revealed differences between genders on measures of negative affect but not of positive affect. This might be explained by the nature of the meditation training, which entailed practices designed to engender concentration and awareness so as to develop equanimity in the face of all emotions and experiences. This is in contrast to practices used to cultivate positive states like self-compassion or loving-kindness toward others, found in other meditation-based clinical programs (Hofmann et al., 2011). It is therefore conceivable that our training would result in decreases of negative emotions but not increases of positive emotions.

It is important to also recognize that neither gender showed improvements on positive affect and the group as a whole did not show improvements on either positive or negative affect. This contradicts other indications of improved positive and negative affect following mindfulness training (Ramel et al., 2004; Chambers et al., 2008). Our data suggests that mindfulness training may not yield improvements in positive or negative affect in all samples or all individuals within a sample and that gender may be one predictor of a differential treatment response.

### Limitations

Our particular sample may limit generalizability to a greater population. The sample was drawn from college –age students at a private university, who have a constricted age range and who are likely to have a higher socioeconomic status than the greater population.

Additionally, all students in our sample chose to take courses with meditation training components and therefore had some particular interest in meditation training. It is possible that the effects of meditation training are different in individuals who are intrinsically motivated, and our study is therefore prone to placebo-effect-like confounds driven by self-selection. While our findings have strong implications for school-based mindfulness programs that are required for all students, these points indicate that they may not be completely generalizable to programs with required rather than self-selected participation.

It should also be noted that our sample did show itself to be potentially atypical for it did not display psychological symptom baseline differences between genders previously shown in other studies (Broderick, 1998, 2005). Additionally, as this study depends on self-report questionnaires, there are always issues of validity and accuracy with self-reported measures. This may be magnified by differences in gender, which may be particularly relevant in our study given previous indications that men may underreport symptoms of depression (Sigmon et al., 2005).

Finally, this study is limited by a binary gender framework— participants were only given the option to identify as either male or female. As expressed gender (and biological sex) is better described on a continuum than a binary, further research should use more nuanced measures of gender (Butler, 1990; Lorber, 1996; Wilchins, 1997; Dvorsky and Hughes, 2008). One possible direction is to better understand if coping mechanisms (e.g., externalizing versus internalizing, or distraction versus rumination) are more accurately correlated to degrees of masculinity and femininity. If so, the differential effects of meditation training on affect might be better understood within that framework. Ultimately an ideal psychological treatment should be individualized; further research toward improving these frameworks can help move the field toward more appropriately targeted treatments.

## Conclusion and Future Directions

This study suggests that college-age men and women may have divergent responses to meditation training. Specifically, women benefit more by demonstrating decreased negative affect and improved mindfulness and self-compassion skills. Conversely, men did not show improvements in negative affect, nor did improvements in mindfulness and self-compassion translate to improved affect as it did in women. This is, to our knowledge, one of the first studies to show affective gender differences in response to meditation training. These results have significant implications for the potential of mindfulness training, particularly to help close gaps in psychological wellbeing between men and women. This research also contributes to the general understanding of gender-specific emotion regulation pathways (Thoits, 1991; Bluhm, 2013; Shields, 2013; Joel and Yankelevitch-Yahav, 2014; Meyers-Levy and Loken, 2015).

Our results suggest gender-specific treatment outcomes may become increasingly salient for men, as they may require mindfulness interventions better matched to the particular coping styles they tend to use. It is also important to consider that mindfulness interventions may be contra-indicated for some individuals (Dobkin et al., 2012). Furthermore, these interventions may require greater attention toward the particular ways men may present issues of psychological or emotional distress. This depth of research and improved focus toward gender differences will help move the scientific and medical fields beyond gendered coping expectations and gender binaries and therefore offer individualized healthcare, tailored for greater holistic, psychological and emotional wellbeing.

## Ethics Statement

This study was carried out in accordance with the recommendations of Human Research Protection Program and Institutional Review Board at Brown University with written informed consent from all subjects. All subjects gave written informed consent in accordance with the Declaration of Helsinki. The protocol was approved by the Institutional Review Board at Brown University.

## Author Contributions

RR: study design, data analysis, data interpretation, drafting, and critical revision. JS: data analysis, data interpretation, drafting, critical revision; equal work to first author RR. HRa: data analysis, data interpretation, drafting, critical revision. HRo: study design, study implementation, data interpretation, and critical revision. WB: study design, study implementation, data analysis, data interpretation, drafting, and critical revision.

## Conflict of Interest Statement

The authors declare that the research was conducted in the absence of any commercial or financial relationships that could be construed as a potential conflict of interest. The reviewer DV and handling Editor declared their shared affiliation, and the handling Editor states that the process nevertheless met the standards of a fair and objective review.
